# Supplementary material for: Exploring the Bacterial Community in Aged Fecal Sources from Dairy Cows: Impacts on Fecal Source Tracking
Source: Microorganisms. 2023 Apr 28;11(5):1161. doi: 10.3390/microorganisms11051161 (PMC10223543; doi:10.3390/microorganisms11051161)
Supplement: Supplementary file 1 [file microorganisms-11-01161-s001.zip › Supplementary_Materials_Figures and Table S7.docx]

Submitted to: Microorganisms for the Special Issue "Gut Microbiome of Farm Animals in Health and Disease 2.0".

**Exploring the Bacterial Community in Aged Fecal Sources from Dairy Cows: Impacts on Fecal Source Tracking**

**Megan L. Devane *, William Taylor *, Pierre-Yves Dupont, Bridget Armstrong, Louise Weaver
and Brent J. Gilpin**

Health and Environment Group, Institute of Environmental Science and Research,
Christchurch 8041, New Zealand; pieer-yves.dupont@esr.cri.nz (P.-Y.D.); bridget.armstrong@esr.cri.nz (B.A.); louise.weaver@esr.cri.nz (L.W.); brent.gilpin@esr.cri.nz (B.J.G.)

***** Correspondence: megan.devane@esr.cri.nz (M.L.D.); william.taylor@esr.cri.nz (W.T.)

**Supplementary Figures**


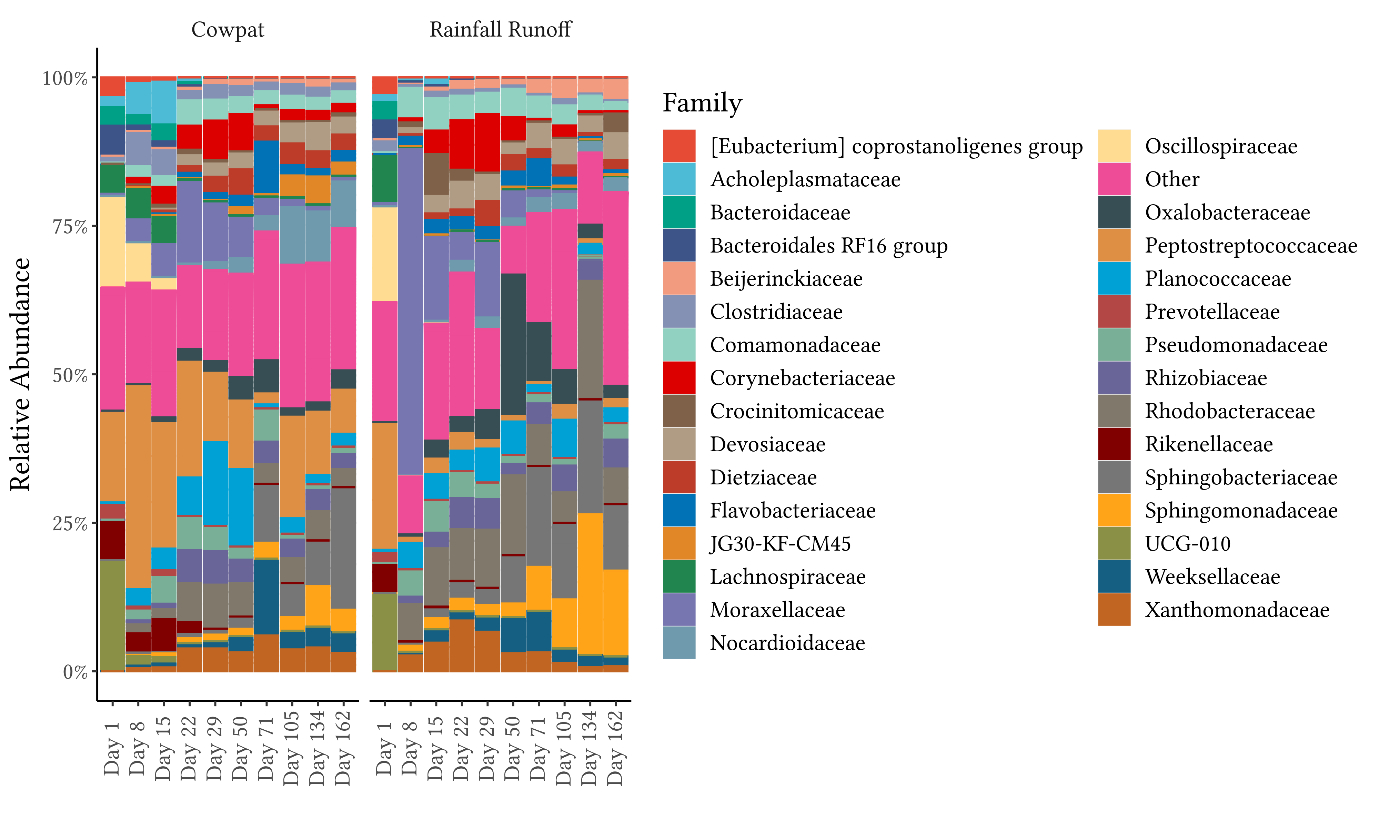


**Supplementary Figure S1.** Relative abundance at the Family level. Cowpat and rainfall runoff samples over time. Bars represent averaged replicates (*n* = 3) at each timepoint, and taxa with average abundances <1% are shown as ‘Other’.


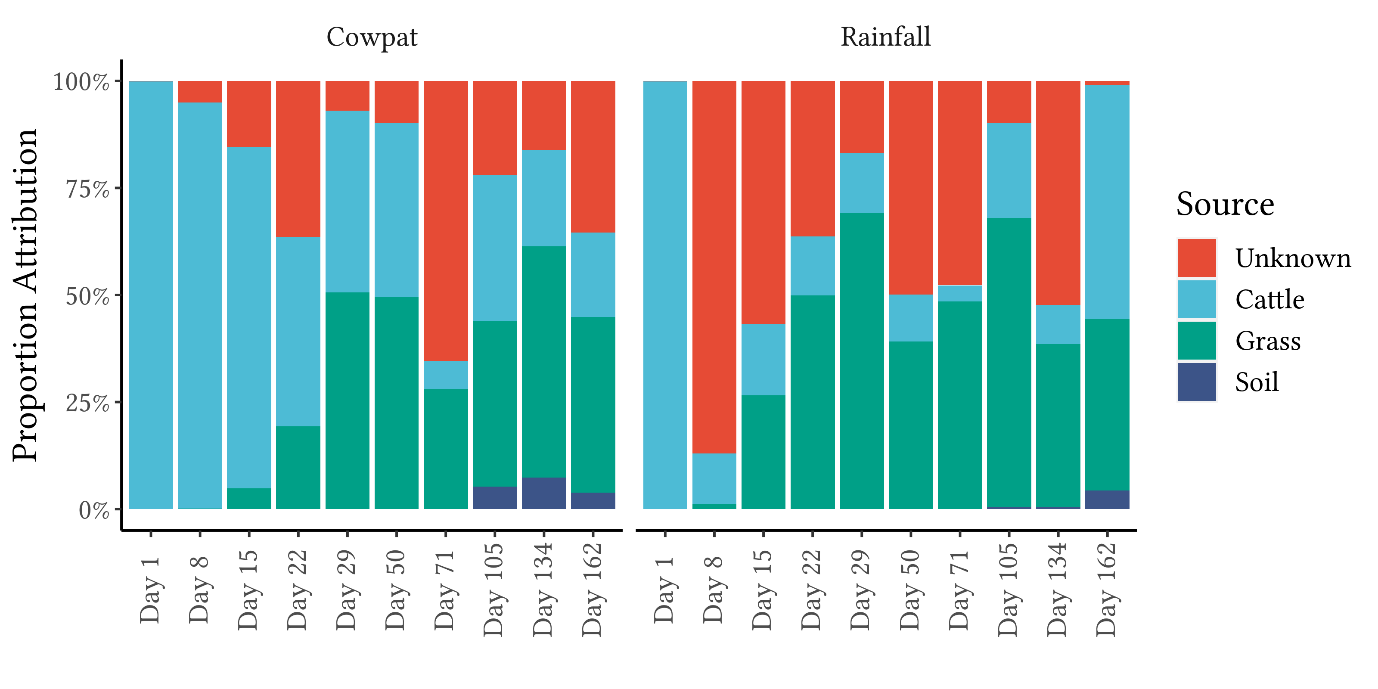


**Supplementary Figure S2.** FEAST output utilizing Class level taxonomy for cowpat and rainfall runoff samples across time.


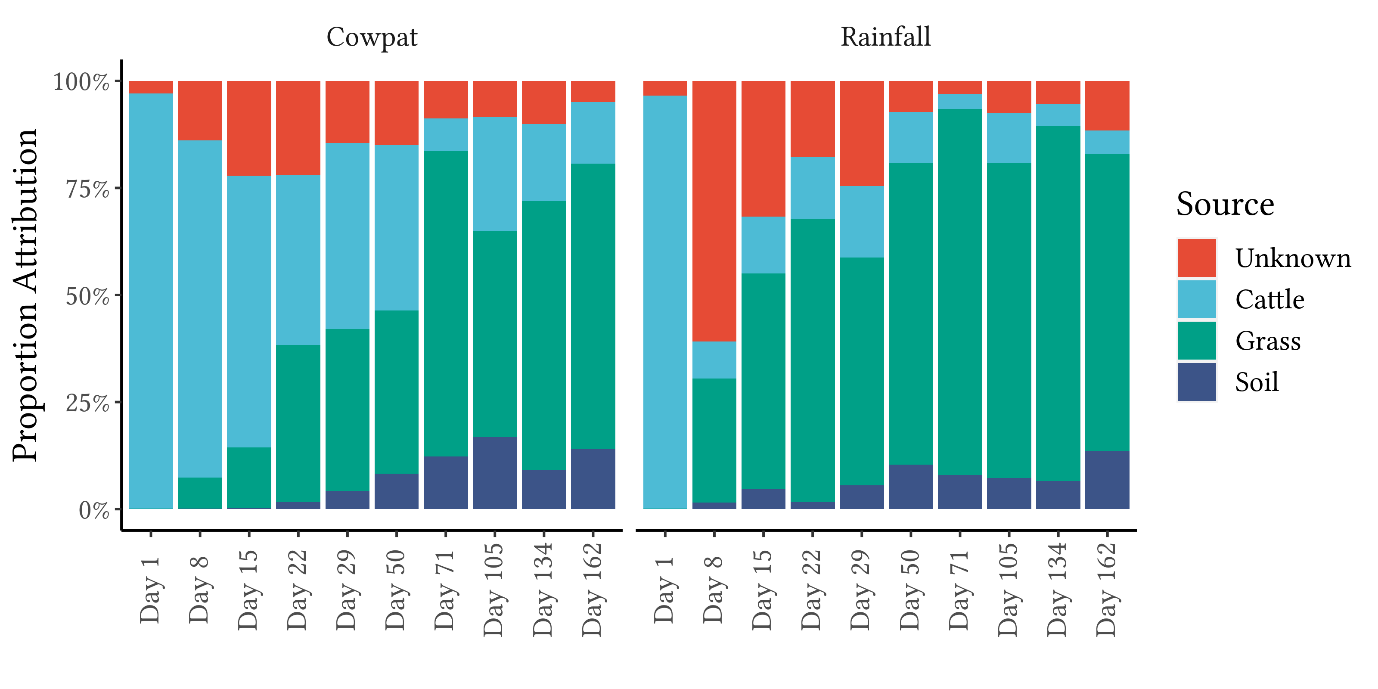


**Supplementary Figure S3.** FEAST output utilizing Family level taxonomy for cowpat and rainfall runoff samples across time.

**Supplementary Table S7.** Top five statistically significant Orders contributing to dissimilarity between selected sampling days.

|  | Cowpat | | | Rainfall Runoff | | |
| --- | --- | --- | --- | --- | --- | --- |
|  | Order | average | p-value | Order | average | p-value |
| Day 1–8 | Oscillospirales | 0.060 | 0.006 | Pseudomonadales | 0.322 | 0.001 |
|  | Bacteroidales | 0.050 | 0.011 | Peptostreptococcales-Tissierellales | 0.296 | 0.001 |
|  | Acholeplasmatales | 0.035 | 0.029 | Oscillospirales | 0.063 | 0.001 |
|  | Campylobacterales | 0.010 | 0.025 | Bacteroidales | 0.044 | 0.001 |
|  | Clostridia vadinBB60 group | 0.003 | 0.006 | Clostridiales | 0.026 | 0.001 |
| Day 1–50 | Corynebacteriales | 0.078 | 0.003 | Peptostreptococcales-Tissierellales | 0.342 | 0.001 |
|  | Bacillales | 0.077 | 0.002 | Burkholderiales | 0.124 | 0.001 |
|  | Oscillospirales | 0.063 | 0.001 | Oscillospirales | 0.074 | 0.001 |
|  | Bacteroidales | 0.045 | 0.014 | Bacteroidales | 0.052 | 0.001 |
|  | Burkholderiales | 0.036 | 0.024 | Clostridiales | 0.032 | 0.001 |
| Day 1–162 | Peptostreptococcales-Tissierellales | 0.221 | 0.001 | Peptostreptococcales-Tissierellales | 0.283 | 0.009 |
|  | Sphingobacteriales | 0.100 | 0.001 | Sphingomonadales | 0.078 | 0.004 |
|  | Oscillospirales | 0.078 | 0.003 | Rhizobiales | 0.073 | 0.005 |
|  | Bacteroidales | 0.072 | 0.001 | Oscillospirales | 0.063 | 0.005 |
|  | Micrococcales | 0.044 | 0.012 | Bacteroidales | 0.045 | 0.004 |
